# Long-Term Sequelae of Retinopathy of Prematurity—A Scoping Review

**DOI:** 10.3390/children13040483

**Published:** 2026-03-30

**Authors:** Philippe Gros-Louis, Tianwei Ellen Zhou, Weronika Jakubowska, Allison L. Dorfman, Anna Polosa, Shigufa Kahn Ali, Valentina Parra, Cynthia X. Qian

**Affiliations:** 1 Department of Ophthalmology, Centre Universitaire d’Ophtalmologie (CUO), Hôpital Maisonneuve-Rosemont, Université de Montréal, Montreal, QC H1T 3W5, Canada; 2 Department of Ophthalmology, Queen’s University, Kingston, ON K7L 3N6, Canada; 3 Department of Ophthalmology, University of British Columbia, Vancouver, BC V6T 1Z4, Canada; 4 Department of Ophthalmology, Centre Hospitalier Universitaire (CHU) Sainte-Justine et Centre de Recherche du CHU Sainte-Justine, Montreal, QC H3T 1C5, Canada; allison.dorfman.hsj@ssss.gouv.qc.ca; 5 Department of Molecular Biology, Medical Biochemistry, and Pathology, Université Laval, Quebec, QC G1V 4G2, Canada

**Keywords:** retinopathy of prematurity, long-term adverse effects, scoping review

## Abstract

**Highlights:**

**What are the main findings?**

- History of ROP signifies a lifelong risk of ocular morbidity.
- Independent effects of gestational age and ROP severity on foveal development remain poorly delineated in the current literature.

**What are the implications of the main findings?**

- Long-term follow-up, including OCT and electrophysiology (fERG, mfERG), should be standard for all affected individuals.
- This ambiguity necessitates future research employing rigorous methodologies to isolate the specific effects of prematurity from those of ROP pathology.

**Abstract:**

**Purpose:** This study aimed to comprehensively map the structural impacts of ROP on all ocular structures, including and extending beyond the inner retina and the associated long-term sequelae that manifest into adulthood. **Methods:** This scoping review identified studies on animal oxygen-induced retinopathy and clinical retinopathy of prematurity using a multi-database search. Study selection and data extraction were performed independently by multiple reviewers using Covidence software. **Results:** ROP results in lasting ocular complications. Posterior segment findings include choroidal insufficiency, photoreceptor dysfunction, and retinal detachment. Anterior segment complications involve a higher incidence of angle-closure glaucoma, strabismus, and significant myopia. **Conclusions:** This scoping review was conducted and reported in accordance with the PRISMA-ScR guidelines, though it is limited by the exclusion of non-English studies. Lifelong ophthalmic monitoring is essential for ROP patients due to persistent anterior and posterior segment complications. This study also identifies key future research priorities, including elucidating mechanisms of foveal development and conducting longitudinal studies. Furthermore, as neonatal intensive care expands in low and middle-income regions, international collaboration is vital to guide screening and treatment and prevent a debilitating surge of ROP.

## 1. Introduction

Retinopathy of prematurity (ROP) is a leading cause of severe visual impairment and blindness among neonates and young children globally. As described by Gilbert [1], the epidemiology of ROP has evolved, with a “third epidemic” now emerging in middle-income countries where improved neonatal survival has increased the population at risk. Traditionally, ROP has been regarded as an inner retinal vasculopathy, ultimately leading to intravitreal neovascularization and potentially resulting in retinal detachment in its most severe form [2]. However, with increasing survival of extremely premature neonates, a growing awareness of progressive dysfunctions within the outer retina is being observed in individuals with a history of ROP, necessitating ongoing follow-up beyond childhood [3]. Determining whether these long-term sequelae stem from ROP severity specifically or from prematurity-related disruptions to normal development, particularly in the fovea, remains an important distinction that will be further explored in this review.

**Figure 1:**
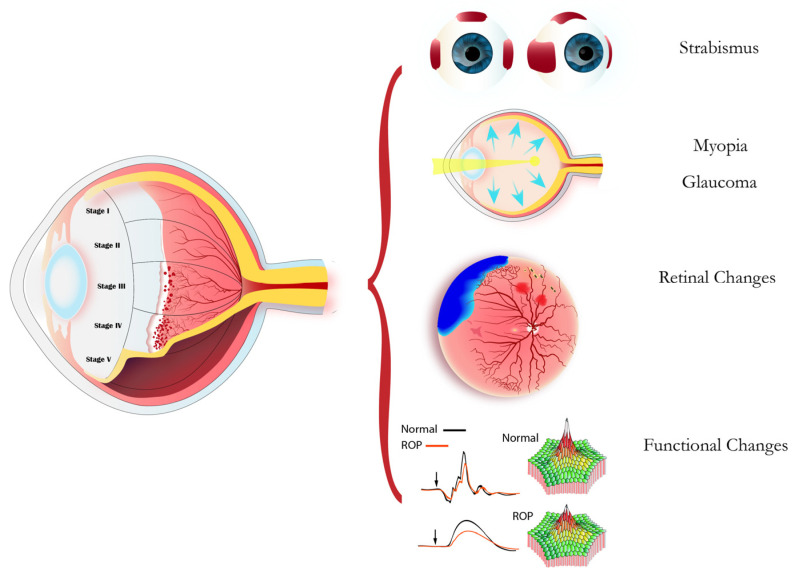
The acute stages of ROP (**left**) progress from a demarcation line (Stage I) to a ridge (Stage II), followed by extraretinal fibrovascular proliferation (Stage III), partial retinal detachment (Stage IV), and total retinal detachment (Stage V). The long-term sequelae (**right**) are categorized into strabismus, myopic shift and glaucoma, sight-threatening retinal changes, and electrophysiologically documented functional retinal (flash ERG; ERG waveforms) and macular (multifocal ERG; 3D topographic plot) changes.

The five-stage classification of ROP, based on the severity of abnormal vessel development at the vascular-avascular retinal junction [4], and its long-term sequelae are summarized in Figure 1.

The initial standard of care for severe ROP was ablative therapy, beginning with cryotherapy to destroy the peripheral avascular retina. Laser photocoagulation later superseded cryotherapy as the preferred method due to its easier application, reduced inflammation, and better long-term structural and visual outcomes [5]. In the past decade, intravitreal anti-vascular endothelial growth factor (anti-VEGF) treatments, such as bevacizumab and ranibizumab, have evolved into the mainstay of treatment for ROP. Although bevacizumab monotherapy has demonstrated effectiveness in managing stage 3 and higher ROP in Zone I [5,6], concerns have been raised by numerous researchers and clinicians regarding its long-term systemic safety, overall efficacy, and potential variations in patient demographics [7]. It has been observed that intraocular administration of bevacizumab can be absorbed into the systemic circulation, leading to suppressed serum VEGF levels for up to 8 weeks in treated premature newborns [8]. This raises concerns about the potential interference with the critical role of VEGF in the development of vital organs.

## 2. Materials and Methods

Animal or human studies reporting structural, vascular, or functional ocular changes associated with retinopathy of prematurity (ROP) or oxygen-induced retinopathy (OIR) were included in this review. The inclusion criteria were peer-reviewed original research articles (including randomized controlled trials, cohort studies, case–control studies, case series and case reports) written in English and studies involving subjects assessed beyond the neonatal stage (i.e., >28 days of life or after reaching term-equivalent age). Studies were excluded if they were editorials, conference abstracts, narrative reviews or if they did not report original data on ocular outcomes. No restrictions were applied based on publication date or the ROP treatments administered.

The study selection process was conducted in two distinct phases: an initial comprehensive search and a subsequent update. For both phases, the title/abstract screening and full-text review were performed independently by at least two reviewers to minimize bias. The screening process was managed using the Covidence systematic review software (https://www.covidence.org/) (Veritas Health Innovation, Melbourne, Australia). Any disagreements at any stage were resolved through discussion or by consultation with a senior reviewer (C.X.Q.).

Initial Search: Two reviewers (A.P., T.E.Z.) independently screened titles and abstracts. Potentially eligible studies then underwent full-text review by three independent reviewers (A.P., T.E.Z., W.J.). The databases searched were MEDLINE and Embase (via Ovid), Scopus, and Web of Science. The initial search was conducted from 1 April to 22 April 2022, and included articles published up to that date.

The search strategy for MEDLINE was developed and then adapted for the other databases using appropriate database-specific syntax and subject headings. The full search string for MEDLINE was as follows:

(“retinopathy of prematurity”[Title/Abstract] OR “retinopathy of prematurity”[Mesh] OR “ROP”[Title/Abstract]) AND (histolog*[Title/Abstract] OR morpholog*[Title/Abstract] OR patholog*[Title/Abstract] OR “Histology”[Mesh] OR “Morphology”[Mesh] OR “Pathology”[Mesh] OR “long-term sequelae”[Title/Abstract] OR “long-term outcome*”[Title/Abstract] OR “chronic complication*”[Title/Abstract] OR “Disease Progression”[Mesh] OR “Chronic Disease”[Mesh]).

**Figure 2:**
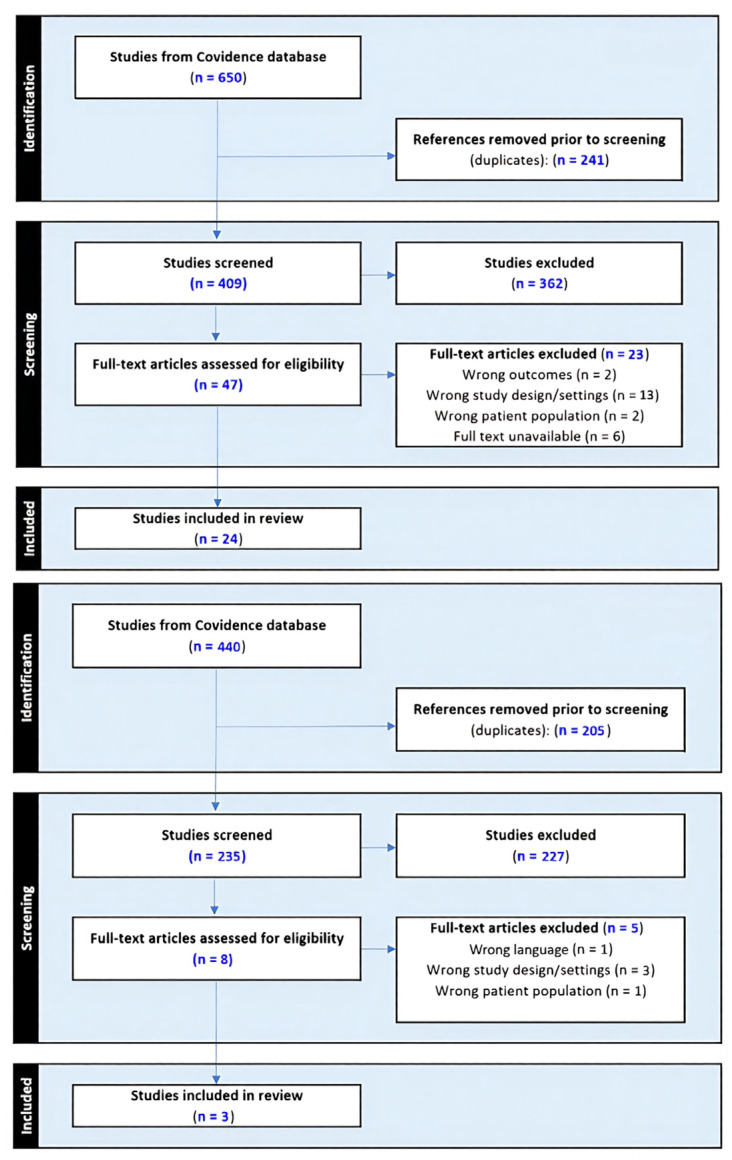
ROP Scoping Review Flowchart Diagrams. Database search in 2022 (**top**) and 2025 (**bottom**) yielded a total of 1090 publications. 446 duplicate references were removed. 644 abstracts were screened, 589 were excluded, and 55 articles met eligibility criteria for full-text assessment. A total of 28 publications were excluded, and 27 studies were included in this review.

Updated Search: The updated search results were screened by two independent reviewers (P.G., M.C.) at the title/abstract stage, followed by full-text review by two reviewers (P.G., A.P.). The databases searched were MEDLINE (via PubMed), Embase, Scopus, ScienceDirect, Cochrane and Web of Science. The updated search was conducted from 17 August to 8 September 2025, and included articles published from 1 January 2022 to 8 September 2025. The study selection process for both phases is detailed in a PRISMA-ScR flow diagram (Figure 2).

Data from the included studies were extracted into a standardized piloted form created in Microsoft Excel (Microsoft Corporation, Redmond, WA, USA). For the initial search, data extraction was performed in duplicate by two independent reviewers (A.P., T.E.Z.). For the updated search, data extraction was performed by a single reviewer (P.G.) and verified by multiple reviewers (S.A., C.X.Q). The characteristics and outcomes of the included studies are summarized in Table A1 (Appendix A).

To ensure methodological rigor, this scoping review adheres to the reporting standards outlined in the PRISMA-ScR (Preferred Reporting Items for Systematic Reviews and Meta-Analyses extension for Scoping Reviews) checklist.

## 3. Results and Discussion

### 3.1. Animal Studies

Animal models have been pivotal in elucidating ROP’s pathogenesis. Following the disease’s initial link to supplemental oxygen [9], Ashton et al. (1953) used a feline model to directly correlate oxygen exposure with ROP vasculopathy, reproducing its hallmark vaso-obliteration and neovascularization [10]. While canine and opossum models followed [11,12], larger animals have become less prevalent due to ethical and practical constraints. The widespread adoption of rodent oxygen-induced retinopathy (OIR) models since the 1990s, notably the Smith (mouse) [13] and Penn (rat) [14] models, is due to their standardization and genetic tractability. However, OIR severity varies significantly by rodent strain [15]. Strain-specific differences and key outcomes in later-discussed rodent OIR models are summarized in Table 1.

#### 3.1.1. Inner Retina

**Figure 3:**
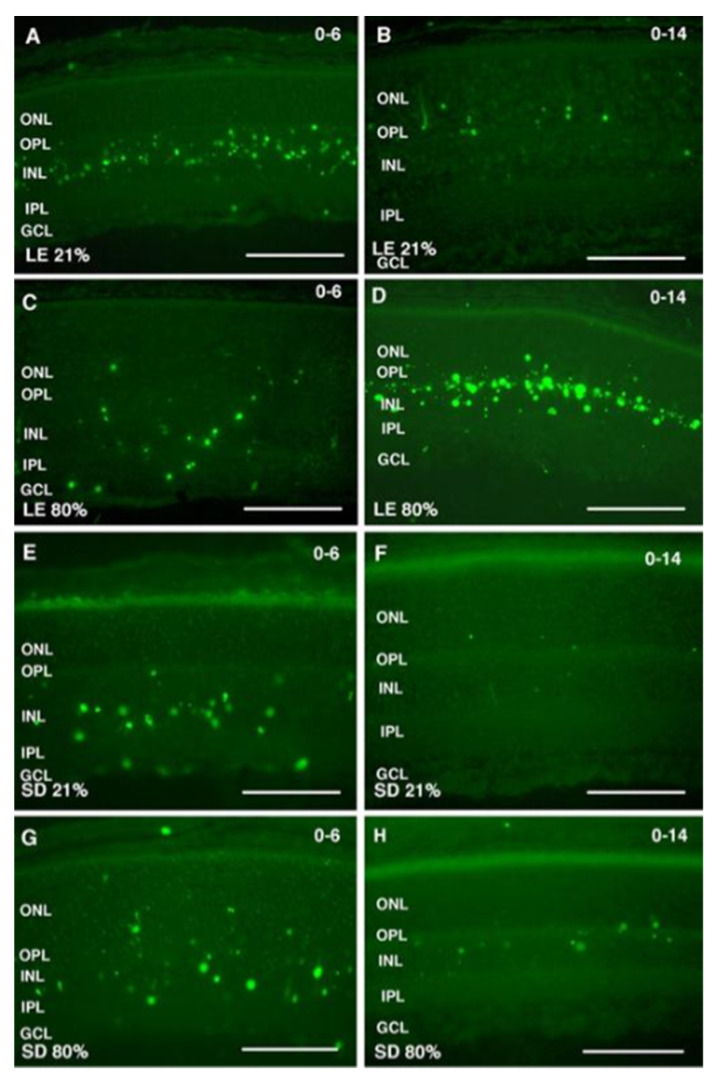
TUNEL staining was performed in Long-Evans (LE) and Sprague-Dawley (SD) rats exposed to hyperoxia for 6 days (P0–P6; (**C**,**G**), respectively) and 14 days (P0–P14; (**D**,**H**), respectively). Data were compared with control LE and SD retinas obtained at P6 ((**A**,**B**), respectively) and P14 ((**E**,**F**), respectively). Scale bars, 100 μm. Reprinted with permission from Investigative Ophthalmology & Visual Science. Originally published in Dorfman et al. [16].

In animal models, ROP’s primary impact on the inner retina is evidenced by an immediate reduction in b-wave amplitude, reflecting vascular obliteration. Histology reveals absent synaptic terminals in the outer plexiform layer (OPL) and disorganized axons in the inner plexiform layer (IPL). With maturation, inner retinal apoptosis increases progressively [16] (Figure 3), and while photoreceptors appear intact, their functional connection to the inner retina is impaired. These experimental findings support the use of electroretinography (ERG) in the clinical follow-up of patients with ROP. ERG may serve as a prognostic tool to detect early signs of disease reactivation, identify those at risk of long-term sequelae, and aid in patient stratification.

#### 3.1.2. Choroid

**Figure 4:**
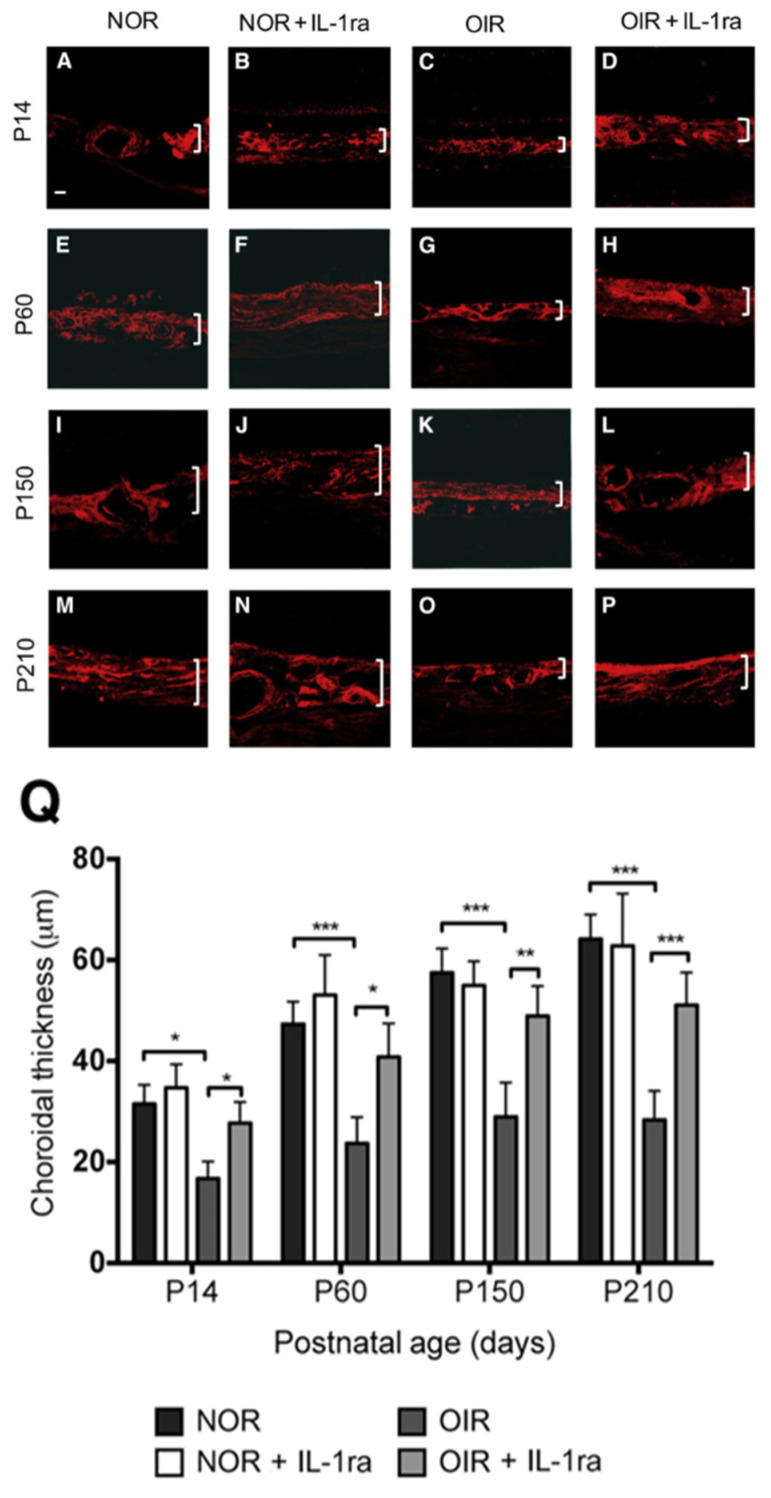
(**A**–**P**): Representative images showing choroidal thickness from animals at different ages (P60, P150, and P210) and following exposure to either cycling oxygen (OIR) or normoxia (NOR), with or without treatment with interleukin-1 receptor antagonist (IL-1ra; from P1 until P14). Choroids were stained with tetramethylrhodamine isothiocyanate–conjugated lectin and images were taken using a confocal microscope. Brackets denote choroidal thickness. (**Q**): Normoxia controls (NOR and NOR + IL-1ra) showed a similar trend in choroidal development. Choroidal thinning (approximately 50%) was detected in the OIR group at all time points investigated; IL-1ra subsequently rescued choroidal involution in OIR animals. Data are expressed as means ± SEM (**Q**). n = 5 to 6 animals (**A**–**P**). * *p* < 0.05, ** *p* < 0.01, and *** *p* < 0.001. Scale bar = 20 μm (**A**–**P**). P, postnatal day. Reprinted with permission from The American Journal of Pathology. Originally published in Zhou et al. [17].

The choroid, which supplies oxygen and nutrients to the outer retina, has been implicated in ROP-associated choroidal insufficiency. In the OIR model, in vitro and ex vivo experiments showed that interleukin-1β (IL-1β), released by retinal pigment epithelium (RPE) cells, exerts cytotoxic effects on choroidal endothelium, resulting in choroidal thinning (Figure 4). These findings support IL-1β as a potential therapeutic target for the treatment of ROP. Longitudinal follow-up of OIR animals throughout their two-year lifespan demonstrated persistent choroidal thinning, leading to photoreceptor layer hypoxia, progressive RPE and photoreceptor loss, and visual dysfunction on ERG [17]. These findings were corroborated by an independent study in young adult OIR mice (postnatal day 47), which reported similar histological and functional impairments [18]. Notably, analogous changes have been observed in patients with a history of ROP (see next section).

### 3.2. Human Studies

#### 3.2.1. Complications of the Posterior Segment

1. Retinal and choroidal anatomical changes

Complications of the posterior segment, such as retinal detachment (RD) and retinal changes, have gained increasing attention as an expanding number of patients with a history of ROP reach adulthood. An observational retrospective series, including 86 eyes from patients born between 1946 and 1964 without ROP treatment during infancy, revealed a notable escalation in the incidence of RD of 25.6% [19]. The Cryotherapy for ROP (CRYO-ROP) randomized clinical trial demonstrated that retinal complications can emerge as late as adolescence: between the ages of 10 and 15, new retinal folds or detachments developed in 4.5% of treated eyes and 7.7% of control eyes [20]. In a more recent multicentre study involving 186 patients with an average age of 34.5 years, it was found that 38.6% of individuals had a RD [21]. Among them, 12.6% had purely tractional RDs and the remaining 86.4% were rhegmatogenous or combined rhegmatogenous/tractional RDs. Notably, in both this study and in the previous literature, the majority of RDs occurred by the age of 30 [21,22]. There are several potential causes of ROP-related RDs. Hamad et al. suggested that the most important mechanisms are likely atrophic hole formation in the avascular retina and residual tractional effects of old fibrovascular membranes [21]. Some clinicians have associated them with the presence of hard exudates, which were believed to originate from telangiectatic retinal vascular abnormalities observed on intravenous fluorescein angiography (IVFA) [23]. More recently, an increasing number of reports have demonstrated a late reactivation of ROP in adolescents and young adults with a prior history of the disease [24,25]. Persistent avascular retina is thought to contribute to late reactivation and subsequent RD through ongoing low-level VEGF expression [24], although both unilateral and bilateral RDs have also been reported in eyes with regressed ROP [26,27].

Apart from RD, other retinal changes are frequently observed in adult patients with a history of ROP. Retinal dragging is one of the prevailing pathological findings in regressed ROP [19]. Eyes that received cryotherapy exhibit a 7-fold higher likelihood of retinal dragging compared to those subjected to laser treatment [28]. The aforementioned multicenter study reported lattice formation in 54.0% of patients, atrophic holes in 34.7%, retinal tears in 30.6%, and tractional retinoschisis in 11.9% of cases. IVFA revealed leakage in approximately half of the cases, while neovascularization occurred in 14.2% of patients [21]. While uncommon, case reports describe late ophthalmic sequelae in ROP patients across different ages, including retinal vessel avulsion in a 59-year-old woman, a vasoproliferative retinal tumor in a 16-year-old, and a spontaneously closed macular hole in a 4-year-old following lens-sparing vitrectomy [29,30,31]. Additionally, a retrospective case series suggested that uncontrolled oxygen therapy in infants weighing ≥1500 g or born at ≥32 weeks’ gestational age (GA) may induce a distinct, severe retinopathy, characterized by widespread non-perfusion and shunting, which differs angiographically from classic ROP and resembles experimental OIR models [32].

Macular thickness is also affected by prematurity and ROP. In a cohort study of 65 children born prematurely and aged 5–16 years, central macular thickness was significantly greater than in age-matched children born at term [33]. In addition, children and young adults with a history of ROP have considerably thicker central maculae than those without ROP [33,34,35].

The evidence regarding foveal hypoplasia in ROP patients is contradictory. Two studies reported a higher prevalence of foveal hypoplasia in patients with a history of ROP [35,36]. In contrast, a third study concluded that lower gestational age was the primary driver of foveal hypoplasia, finding no independent association with ROP after adjusting for prematurity [34]. These results highlight a critical uncertainty in the literature: does ROP actively disrupt foveal development, or are foveal abnormalities simply concurrent sequelae of extreme prematurity?

Resolving this contradiction is essential for understanding long-term visual outcomes in ROP survivors. Although the present scoping review did not perform meta-analysis, the accumulating data, particularly studies reporting standardized OCT metrics, suggest that future meta-analytic synthesis could quantitatively determine the relative contributions of ROP severity and gestational age to foveal structure. Such an approach would enable subgroup analyses stratified by ROP stage, treatment history, and postnatal age, potentially clarifying whether foveal hypoplasia represents a direct disease effect or a developmental consequence of prematurity.

Along with foveal hypoplasia, Nilsson et al. documented two associated microstructural anomalies in ROP-adults treated with cryotherapy: persistent inner retinal layers (IRL) at the foveal center and an increased thickness of the temporal retinal nerve fiber layer (RNFL) [36].

In addition to retinal alterations, changes in choroidal thickness have been documented in the context of ROP. Several studies utilizing optical coherence tomography (OCT) have reported choroidal thinning in older children and young adults with a history of ROP [37,38,39]. A key finding of this review is the significant choroidal thinning (≥15%) observed in former ROP patients, regardless of spontaneous resolution [39]. However, accumulating evidence suggests that the choroidal vascular index (CVI), a measure of vascular density derived from OCT image binarization, may be a more reliable and informative parameter than thickness alone for elucidating the choroid’s association with ROP [40,41]. Quantitatively, Oruz and Dervişoğulları reported significantly reduced CVI in preterm children (69.58 ± 2.72%) compared to term-born controls (71.90 ± 2.60%) (*p* < 0.001), while choroidal thickness did not differ significantly between groups [40]. Similarly, Lavric et al. found that CVI was significantly lower in preterm children with a history of ROP (68.66 ± 3.24%) versus full-term controls (71.37 ± 3.63%) (*p* = 0.005), with no corresponding difference in subfoveal choroidal thickness [41]. These findings indicate that CVI detects vascular compromise even when thickness measurements appear normal, supporting its role as a sensitive biomarker for ROP-related choroidal injury. Consequently, ROP is not solely an inner retinal vasculopathy but also a choroidopathy, affecting the primary blood supply to the RPE and photoreceptors.

2. Functional changes

In addition to the vascular and structural sequelae described above, ROP has also been associated with deficits in retinal function.

While prematurity alone, in the absence of ROP, appears to have little effect on rod function, both mild and severe ROP are associated with significant impairment of rod photoreceptor function, as well as post-receptor dysfunction [42]. Some evidence suggested that patients with mild ROP may benefit from substantial reorganization and rewiring of post-receptor retinal circuitry, similar to that proposed in animal models of ROP [3,15,17]. It was later shown in subjects with mild ROP that although both rod photoreceptor and post-receptor sensitivities were affected in infancy, post-receptor function recovered by childhood [42]. In contrast, the severe ROP group revealed lasting deficits in both rod photoreceptor and post-receptor sensitivities. The cone system, however, appears to be less vulnerable to the adverse effects of ROP. Fulton et al. [43] attributed cone resistance to their earlier maturation and to anatomical and metabolic properties that confer greater protection from fluctuations in oxygen levels and the resulting oxidative stress compared to the rod system. Other studies also confirmed these observations in school-aged children [33,44].

On the full-field flash ERG (fERG) which measures the pan-retinal function of the rod and cone pathways, changes in ROP patients are reflected by abnormal amplitude and peak times values. Overall, the literature suggests that both the a-wave and the b-wave of the fERG response, as well as some dark-adapted oscillatory potentials, can be affected in eyes with ROP, with greater reductions in amplitude observed in more severe cases [45,46,47].

Studies using multifocal ERG (mfERG) in older ROP patients have revealed central retinal dysfunction, notably reduced response amplitudes. Ecsedy et al. hypothesized this results from a central disparity in bipolar cell density [44], which was later corroborated by another report of abnormal mfERG peaks in an ROP patient [48].

Persistent functional anomalies have also previously been described in a relatively younger cohort as well, namely school-aged children with a history of ROP. Despite resolution of the vascular component, these patients had lasting functional sequelae as documented with both the fERG [43,44,49] and the mfERG [50,51,52,53].

#### 3.2.2. Complications of the Anterior Segment

1. Glaucoma

Angle-closure glaucoma is a recognized complication of ROP and occurs in approximately 1–2% of patients [54,55]. The narrow angle in adults with a history of ROP can result from iris or angle neovascularization, retrolental mass, pupillary block, choroidal congestion, or anterior displacement of the ciliary body [56,57]. While improved neonatal care has drastically reduced severe sequelae like angle closure from retrolental masses or neovascular glaucoma, former ROP patients who develop non-neovascular angle closure do so decades earlier (average 33 years) than those with primary angle closure [56].

A large-scale retrospective study examined 407 eyes of 213 patients with ROP stages 0 to 5 [58]. Over a follow-up of 69 years, the authors reported that 38.1% of eyes developed ocular hypertension (OHT). Subgroup analyses revealed a significantly higher incidence of OHT in eyes that had undergone acute incisional retinal surgery. Furthermore, among stage 4 and stage 5 eyes, phakic eyes were associated with a significantly lower OHT incidence compared with aphakic eyes. These data underscore the increased lifelong risk of adverse ocular sequelae in patients with a history of prematurity, especially those who had surgical interventions.

2. Cornea

The impact of ROP on the cornea is indirect, often as a result of its retinal complications [54,55] and treatments [59]. For instance, corneal opacity and irregularity secondary to multiple surgeries can potentially precipitate amblyopia [55]. Fieß et al., in their 2022 retrospective cohort study, demonstrated that ROP was not associated with thinner corneal thickness [60]. As such, efforts should be directed to maintaining a healthy and normal corneal contour when caring for this population.

3. Strabismus

ROP significantly increases strabismus risk [61]:

- Full-term infants: 0.5–9.9%
- Preterm infants without ROP: 17.4%
- Preterm infants with untreated ROP: 27.1%
- Preterm infants with treated ROP: 60%

Notably, even extremely preterm individuals without ROP exhibit increased visual deficits, suggesting contributions from prematurity-related factors. While ROP status modifies the severity and type of deficits, it does not fully explain the elevated risk, underscoring the multifactorial nature of prematurity-associated visual morbidity [62].

Of note, eyes previously subjected to cryotherapy for ROP exhibited an increased risk of macular ectopia (2.7%), resulting in pseudostrabismus. In a case series of patients aged 6 to 26 years with macular ectopia following acute ROP, esotropia and pseudoexotropia occurred simultaneously [63]. Macular ectopia may be accompanied by an esodeviation with early-onset strabismus, which can partially mask an underlying divergent pseudoexotropia.

Management considerations: Although strabismus resulting from ROP sequelae may share certain features with congenital strabismus, a more conservative management approach is recommended. In cases involving combined esotropia and pseudoexotropia secondary to macular ectopia, surgical intervention is not advised, as the misalignment is partly anatomical rather than purely strabismic. Clinicians should maintain a high index of suspicion for pseudostrabismus in ROP survivors to avoid unnecessary surgery.

#### 3.2.3. Other Changes

1. Refractive Changes

Adults with a history of ROP during infancy exhibit a significantly higher prevalence of myopia and refractive errors. Smith et al. reported that nearly 90% of individuals in this cohort had some degree of myopia, with a mean refractive error of −5.71 diopters and 75% of eyes exhibiting moderate to high myopia [19]. Within this untreated ex-ROP adult group, approximately half of the subjects displayed a best-corrected visual acuity (BCVA) of 20/200 or lower, and one-third had vision limited to counting fingers or worse. Kaur et al. also reported an increased prevalence of myopia and other refractive errors in older children with ROP [64].

While both laser photocoagulation and cryotherapy increase the risk of myopia and peripheral vision loss [65], long-term data show laser treatment yields superior visual outcomes, including less myopia and a five-fold greater likelihood of achieving a BCVA of 20/50 or better [28]. In contrast, anti-VEGF therapy (e.g., bevacizumab, ranibizumab) for ROP is associated with significantly lower rates of myopia and high myopia compared to laser treatment, thereby offering superior refractive safety [66,67]. In ROP, myopia progression is primarily driven by increased lens thickness and power, whereas in full-term myopia, axial length is the main contributor [68].

Although landmark studies such as Cryo-ROP [69], ET-ROP [70], and BEAT-ROP [6] were instrumental in establishing treatment guidelines and improving outcomes for ROP patients, they also underscore the considerable shifts in modern clinical management. Consequently, the relevance of some existing literature is limited, as certain studies are based on now-outdated treatment modalities. Moreover, drawing definitive conclusions from the current body of evidence is challenging. Data derived from surgical case series are often subject to selection bias, and the true population-wide incidence of late retinal detachment remains unknown.

This review has several limitations. First, as a scoping review, we did not formally assess the methodological quality or risk of bias of the included studies, which precludes the formulation of clinical recommendations based on evidence strength. Second, restricting inclusion to English-language publications may have introduced language bias, potentially underrepresenting regional data and overrepresenting findings from North American and European populations, thereby limiting generalizability. Third, the possibility of publication bias cannot be excluded, as studies reporting positive or significant findings are more likely to be published. Finally, the heterogeneity of study designs, outcome measures, and animal models included in this review limited direct comparisons across studies.

## 4. Conclusions

This scoping review identified 27 studies characterizing the long-lasting effects of ROP on the anterior and posterior segments, as well as the structural and functional impacts on animal and human eyes. The findings confirm that a history of ROP signifies a lifelong risk of ocular morbidity. Consequently, a structured long-term follow-up protocol, inclusive of OCT and electrophysiological testing (fERG, mfERG), should be standard for all affected individuals. These functional assessments could detect subclinical retinal dysfunction before structural damage is evident on imaging, enabling early risk stratification and timely intervention to preserve long-term visual function. However, we acknowledge the practical challenges associated with their implementation, including the need for specialized equipment, patient cooperation, and the burden on patients and healthcare systems. Subsequent studies should aim to establish practical, risk-stratified follow-up algorithms that balance clinical utility with feasibility.

Further research should delineate the distinct mechanistic pathways by which gestational age and ROP severity independently influence foveal development and clarify their relative contributions to structural and functional outcomes. Concurrently, large-scale longitudinal studies following preterm infants into adulthood are needed to establish incidence rates and natural history trajectories for ROP-related complications.

As neonatal intensive care capacities expand in low and middle-income regions, including many African countries, a new surge in ROP is anticipated. Leveraging experiences from European and North American cohorts through international collaboration will be essential to help guide ROP screening, treatment and strategies to prevent and address the debilitating long-term sequelae of ROP.

## Figures and Tables

**Table 1:** Strain-specific differences and key outcomes in rodent OIR models [16,17,18].

Species	Strain	Key Outcomes
Rat	Sprague-Dawley (albino) and Long-Evans (pigmented)	Progressive RPE and photoreceptor loss Visual dysfunction on ERG Progressive choroidal thinning Inner retinal apoptosis and synaptic disorganization
Mouse	C57BL/6J (pigmented)	Progressive RPE and photoreceptor loss Visual dysfunction on ERG IL-1β-mediated choroidal injury

## Data Availability

No new data were created or analyzed in this study.

## Abbreviations

The following abbreviations are used in this manuscript:

ROP	Retinopathy of prematurity
Anti-VEGF	Anti-vascular endothelial growth factor
OIR	Oxygen-induced retinopathy
OPL	Outer plexiform layer
IPL	Inner plexiform layer
RPE	Retinal pigment epithelium
ERG	Electroretinography
RD	Retinal detachment
IVFA	Intravenous fluorescein angiography
GA	Gestational age
IRL	Inner retinal layers
RNFL	Retinal nerve fiber layer
OCT	Optical coherence tomography
CVI	Choroidal vascular index
fERG	Flash ERG
mfERG	Multifocal ERG
OHT	Ocular hypertension
BCVA	Best-corrected visual acuity

## Appendix A

**Table A1:** Characteristics and outcomes of the 27 included studies.

Author (Year)	Study Design	Population (Age)	Follow-up Period	Key Outcomes	Major Findings	Limitations
Altschwager et al. (2017) [53]	Case–Control	40 (9–17)	N/A	MfERG amplitude/implicit time, localized retinal function in preterms	Preterm birth reduces central amplitudes, indicating persistent inner retinal dysfunction	Unable to distinguish effects of prematurity, ROP and treatment, small severe ROP sample
Akerblom et al. (2011) [33]	Cross-sectional	120; 29 ROP (5–16)	N/A	Retinal macular thickness in preterms	Thicker central macula in preterm children is linked more strongly to their degree of prematurity than to a history of ROP	Confounding bias, small severe ROP sample, unable to evaluate individual retinal layers with OCT technique used
Bariya et al. (2024) [31]	Case report	1 infant	4 years	Late macular lesion after lens-sparing vitrectomy for ROP	Spontaneously closed macular hole, potentially due to tangential traction from the residual posterior hyaloid	Small sample, single center, lack of sequential follow-up
Bianchi et al. (1996) [63]	Case series	5 (6–26)	N/A	Esotropia and pseudoexotropia in ROP	Pseudoexotropia from ROP is true esotropia, requiring correct identification to avoid mistreatment	Retrospective design, small sample, no control group
Brown et al. (1994) [23]	Case series	11 (22–39)	N/A	Exudative retinopathy	A potential late sequela of ROP	Retrospective design, small sample, no control group
Chow et al. (2011) [30]	Case report	1 (16)	3 years	Vasoproliferative retinal tumor	A potential late sequela of ROP	Small sample, Single center
Czeszyk et al. (2024) [35]	Cross-sectional	123; 52.5% eyes ROP (IQR: 8.12–12.77)	N/A	Macular morphology and vessel density of preterms	Smaller foveal avascular zone, higher vessel density, increased foveal thickness and thinner choroid in ROP, the last two being strongly associated with laser photocoagulation therapy	Selection bias, information bias, small sample for aggressive ROP group
Ecsedy et al. (2007) [44]	Case–control	40; 20 ROP (7–14)	N/A	Macular structure and thickness in preterms	Thicker central foveal region and shallower foveal depression	Single-center, single time point, small sample
Fieß et al. (2022) [34]	Retrospective cohort	390; 39 ROP (21–38)	N/A	Corneal thickness (apex, pupil center, periphery)	ROP is not associated with thinner corneal thickness	Single-center, non-response bias, measurement bias, small ROP sample
Fledelius and Jensen (2011) [48]	Case series	31; 15 ROP (6–58)	4 to 58 years	Late anterior and posterior segment complications or visual loss in preterms	Ocular vulnerability from prematurity predisposes to late complications, regardless of initial ROP severity	Retrospective design, small sample, lack of Standardized follow-up, heterogeneous cohort
Golas et al. (2018) [24]	Case report	1 (19)	N/A	Retinal detachment	A potential late sequela of untreated ROP	Small sample, no control group, single center
Hamad et al. (2020) [21]	Case series	186 (7–76)	N/A	RD and its risk factors in non-treated ROP	High rate (38.6%) of RD, often associated with persistent avascular retina, atrophic holes and vitreous traction	Retrospective design, anterior segment pathology/amblyopia not reported
Hansen et al. (2017) [42]	Prospective cohort	100 to 300 (0.1–60)	Variable	Neural retinal development in ROP	- Preterm birth alone minimally affected rod function - ROP induce lasting neurosensory retinal deficits, including postreceptoral dysfunction	Single-center, attrition bias, preclinical bias
Harris et al. (2011) [3]	Cross-sectional	50 (3–23) + 35 infants; 64 ROP	N/A	Rod-mediated ERG responses and rod-driven pathway function	In mild ROP, beneficial reorganization of post-receptor retinal circuitry improves ERG sensitivity and normalizes visual thresholds	Focus on Rods Only, Single time point
Jain et al. (2022) [62]	Prospective cohort	128; 52% eyes ROP (19)	19 years	Visual function and ocular morbidity in extreme prematurity	Extreme prematurity is linked to worse BCVA, strabismus, and nystagmus, often independent of neonatal ROP status	Subject loss, healthy participant bias
Kaur et al. (2017) [64]	Cross-sectional	36 (6–15)	N/A	Long-term refractive error, biometry and VA in laser-treated ROP	- Lower GA is associated with larger refractive errors and lower VA - Higher risk of myopia/greater axial length/high astigmatism in ROP	Retrospective design, no control group, Incomplete longitudinal data, sampling bias
Lin et al. (2021) [27]	Case report	1 (36)	4 months	Bilateral retinal detachment	A potential late sequela of untreated ROP	Small sample, no control group, single center
Martinez-Castellanos et al. (2017) [32]	Retrospective case series	14 infants	N/A	Peripheral vascular changes on FA	Larger preterms (>32 wks GA & >1500 g) exposed to poorly controlled high oxygen develop a retinopathy distinct from classic ROP	Retrospective design, small sample, no control group
Mezu-Ndubuisi et al. (2020) [18]	Experimental longitudinal animal model	45 mice; 22 OIR	28 days	Functionality and anatomy of mice with OIR	Persistent retinal dysfunction and structural abnormalities in adulthood, despite partial vascular recovery	Species differences, model specificity, translation to humans
Nilsson et al. (2016) [36]	Retrospective case–control	27; 14 ROP (22–26)	N/A	Long-term structural retinal changes in cryotherapy treated ROP	Reduced foveal depth/absence of foveal pit, incomplete extrusion of the IRL at the fovea center and increased thickness of the temporal RNFL	Small sample, selection bias, single-center study
Robinson et al. (2018) [58]	Retrospective cohort	213 (15–69)	Up to 35.7 years (Mean 14.3)	Ocular hypertension in ROP stage 0 to 5	Adults born prematurely face a high, lifelong risk of OHT, which increases with ROP severity and is greatest after surgical intervention, particularly lens removal.	Retrospective design, referral and selection bias, confounding variables
Smith & Tasman (2005) [19]	Retrospective case series	47 (45–56)	N/A	Prevalence of late ocular complications in ROP without treatments	High rate of posterior segment pathology, RD is common and occurs late, high prevalence of cataracts and myopia, risk of glaucoma, decreased BCVA	Retrospective design, no control group, selection bias
Tarafdar et al. (2011) [29]	Case report	1 (59)	N/A	Structural complication of ROP	Avulsed retinal vessels secondary to posterior vitreous detachment	Small sample, no control group, single center
Terasaki & Hirose (2003) [26]	Retrospective case series	29 (6–51)	1–26 years	Anatomical success of surgery and postoperative VA of late-onset RD in regressed ROP	- High surgical success rate anatomically but poor functional vision outcomes. - High risk in the fellow eye	Retrospective design, small sample, no control group, selection bias
Tufail et al. (2004) [22]	Retrospective case series	32 (8–59)	2 months-12 years	Anatomical success of surgery, postoperative VA and prophylactic treatment success in complications of regressed ROP	- High final success rate with multiple procedures - No single best surgical technique - Significant visual improvement - Prophylactic treatment is effective	Retrospective design, no control group, non-standardized treatment
Uner et al. (2020) [25]	Retrospective case series	5 (13–43)	1–7 years	Status of exudation and vasoproliferation	Exudation and fibrovascular proliferation can develop later in some adolescents and adults with regressed ROP (3.6%)	Retrospective design, small sample, referral bias, No control group, incomplete neonatal records
Zhou et al. (2016) [17]	Experimental longitudinal animal model	OIR rat	210 days	Long-term preservation of visual function	Early IL-1β inhibition preserves choroidal health, preventing hypoxia and subsequent degeneration of RPE and photoreceptors to improve lifelong visual function	Species differences, model specificity, translation to humans

BCVA, Best-corrected visual acuity; FA, Fluorescein angiography; GA, Gestational age; IQR, Interquartile range; IRL, Inner retinal layers; MfERG, multifocal electroretinogram; OHT, Ocular hypertension; OIR, Oxygen-induced retinopathy; RD, Retinal detachment; RNFL, Retinal nerve fiber layer; ROP, Retinopathy of prematurity; VA, Visual acuity.
